# A Glutathione Peroxidase, Intracellular Peptidases and the TOR Complexes Regulate Peptide Transporter PEPT-1 in *C. elegans*

**DOI:** 10.1371/journal.pone.0025624

**Published:** 2011-09-28

**Authors:** Jacqueline Benner, Hannelore Daniel, Britta Spanier

**Affiliations:** ZIEL Research Center of Nutrition and Food Sciences, Abteilung Biochemie, Technische Universität München, Freising, Germany; New Mexico State University, United States of America

## Abstract

The intestinal peptide transporter PEPT-1 in *Caenorhabditis elegans* is a rheogenic H^+^-dependent carrier responsible for the absorption of di- and tripeptides. Transporter-deficient *pept-1(lg601)* worms are characterized by impairments in growth, development and reproduction and develop a severe obesity like phenotype. The transport function of PEPT-1 as well as the influx of free fatty acids was shown to be dependent on the membrane potential and on the intracellular pH homeostasis, both of which are regulated by the sodium-proton exchanger NHX-2. Since many membrane proteins commonly function as complexes, there could be proteins that possibly modulate PEPT-1 expression and function. A systematic RNAi screening of 162 genes that are exclusively expressed in the intestine combined with a functional transport assay revealed four genes with homologues existing in mammals as predicted PEPT-1 modulators. While silencing of a glutathione peroxidase surprisingly caused an increase in PEPT-1 transport function, silencing of the ER to Golgi cargo transport protein and of two cytosolic peptidases reduced PEPT-1 transport activity and this even corresponded with lower PEPT-1 protein levels. These modifications of PEPT-1 function by gene silencing of homologous genes were also found to be conserved in the human epithelial cell line Caco-2/TC7 cells. Peptidase inhibition, amino acid supplementation and RNAi silencing of targets of rapamycin (TOR) components in *C. elegans* supports evidence that intracellular peptide hydrolysis and amino acid concentration are a part of a sensing system that controls PEPT-1 expression and function and that involves the TOR complexes TORC1 and TORC2.

## Introduction

Dietary proteins within the intestinal lumen are hydrolyzed to oligopeptides which in turn get cleaved to di- and tripeptides and free amino acids by membrane anchored peptidases of intestinal brush border membranes [1]. Amino acid transporters are responsible for the uptake of free amino acids while a large portion of amino acids are taken up as di- and tripeptides by the intestinal peptide transporter PEPT1 (SLC15A1) [2]. Peptide transport across cell membranes takes place in all living organism. PEPT1 exhibits a broad substrate specificity and transports with a few exceptions, around 400 dipeptides and 8000 tripeptides that result from digestion of dietary and body protein [3]. In addition, PEPT1 also enables the absorption of drugs such as aminocephalosporins, anticancer drugs or antiviral agents like acyclovir [4], [5]. PEPT1 is an electrogenic symporter that couples substrate transport to proton movement across the membrane therefore leading to an acidification of the cytosol. The driving force for this transport is the inwardly directed H^+^-electrochemical gradient and membrane potential that allows substrate accumulation to concentrations above extracellular levels [6]. For the maintenance of the proton gradient and intracellular pH homeostasis, the sodium-proton-exchanger NHE3 (SLC9A3), named NHX-2 in *C. elegans,* is required [6], [7], [8].

In *C. elegans*, three peptide transporter isoforms are found [9] namely PEPT-1 (OPT-2, PEP-2), PEPT-2 (OPT-1, PEP-1) and PEPT-3 (OPT-3). The *C. elegans pept-1* gene encodes a protein that is similar to the low-affinity, high-capacity isoform designated as PEPT1 in mammals with prominent expression in the intestine [9]. PEPT-1 exhibits 36.9 % sequence homolgy with the human PEPT1 and gene deletion in the worms abolishes intestinal peptide uptake [10]. The requirement of the sodium-proton antiporter NHX-2 for peptide transporter function and recovery from intracellular acid load has been demonstrated in *C. elegans* [11]. PEPT-1 deficiency in the nematode causes decelerated larval development, increased reproductive lifespan, smaller body size, reduced brood size, enhanced stress resistance [10] and an obese phenotype [12]. The high body fat content in *pept-1 C. elegans* is driven by a decreased proton influx followed by an alkalization of the intestinal cells which promotes the uptake of free fatty acids. On the contrary, the loss of NHX-2 decreases the proton efflux and promotes the intracellular acidification by the PEPT-1 proton-dipeptide-symport which finally reduces fatty acid uptake and induces a lean phenotype [12].

In mammals, PEPT1 expression is found to be regulated by diet, developmental stage of the organism and certain hormones. High-protein diets, thyroid hormone, epidermal growth factor and leptin induce PEPT1-mRNA expression and/or mRNA-stability and insulin seems to directly increase the membrane population of PEPT1 by promoting its trafficking to the apical membrane [for review see 13]. For the PEPT2 isoform, the serum/glucocorticoid inducible kinase SGK1 as well as the NHE3 regulatory factor, NHERF1 were found to be modulators at the post-translational level [14]. However, the present knowledge of proteins that modulate PEPT1 function and transport activity either directly or indirectly is rather limited and scarce.

In this study, we describe the identification of such modulators for PEPT-1 function in the model organism *C. elegans*. Gene silencing of four genes coding for gluthatione peroxidase, an ER to Golgi transport protein and two amino peptidases modified the protein expression and transporter function of PEPT-1. We found strong evidences that these functional modulations are also conserved in the human Caco-2/TC7 colon carcinoma cell line. Our work also identifies a novel sensing pathway which controls membrane expression of the PEPT-1 protein and consequently peptide absorption capacity.

## Materials and Methods

### *C. elegans* strains and nematode culture

The following *C. elegans* strains were used: wildtype N2 Bristol, *pept-1*(*lg601*) (BR2742), P*pept-1::GFP;rol-6(su1006)* (BR2875) and *rrf-3*(*pk1426*) (NL2099). All *C. elegans* strains were grown and maintained at 20°C as a mixed population on nematode growth medium (NGM) agar plates seeded with *E. coli* OP50 [15]. For RNAi experiments, mixed stage *rrf-3*(*pk1426*) *C. elegans* were fed for seven days with the corresponding *E. coli* strains from Julie Ahringers *C. elegans* RNAi library [16], [17] following the protocol described earlier [18]. As a control, *rrf-3(pk1426) C. elegans* were grown on *E. coli* HT115 containing only the empty vector pPD129.36 (L4440) (following called vector control, vc(RNAi)). To synchronize *C. elegans* populations, eggs were prepared by hypochlorite treatment.

### Transport assay employing the fluorescent dipeptide ß-Ala-Lys-AMCA (RNAi Screen)

Synchronized *rrf-3(pk1426)* L1 larvae were grown for three days on vc(RNAi) or on plates seeded with RNAi bacteria producing double-strand RNA (dsRNA) of *pept-1* (additional control) or one of the 162 intestinal expressed genes (RNAi clones) until control worms reached the L4 larval stage. As an additional control, synchronized *pept-1* knockout L1 larvae were grown for five days on vc(RNAi) until they also reached the L4 larval stage. Since it was not possible to analyze all of 162 RNAi clones at one time, several experiments were set up and controls (*rrf-3*;vc(RNAi), *rrf-3;pept-1*(RNAi), *pept-1*;vc(RNAi) and *rrf-3*;vc(RNAi) without the addition of ß-Ala-Lys-AMCA) were included in each experiment. The L4 larvae were washed off the RNAi plates with M9 buffer. A final concentration of 0.25 mM ß-Ala-Lys-AMCA was added to the samples and they were incubated for 3 hours at 20°C. 0.5 ml liquid *E. coli* OP50 culture was added and the samples incubated for an additional hour to wash the ß-Ala-Lys-AMCA out of the intestinal lumen. After 4–6 washing steps with M9 buffer, the nematodes were placed on empty agar plates and photographed for body length analysis. Afterwards, 20 nematodes were transferred into one well of a black 384-well plate. For each control/RNAi clone four replicates were performed. Fluorescent signals were detected (excitation 340 nm, emission 445 nm) using a multimode plate reader (Thermo Electron Corporation, USA). The calculation of relative ß-Ala-Lys-AMCA-uptake was done as follows. After subtraction of the auto fluorescence, each value was multiplied by the relative body length of the nematode with the length of *rrf-3*;vc(RNAi) set to one. Then the mean fluorescence of *pept-1*;vc(RNAi) worms was subtracted from each value. Relative ß-Ala-Lys-AMCA uptake was calculated in relation to *rrf-3*;vc(RNAi). Each experiment was repeated at least twice. Significance was calculated using the Student's *t*-Test and the result of each of the 162 RNAi clones was solely compared with the control.

### Microscopic analysis of the PEPT-1 transporter function

To individually analyze the uptake of ß-Ala-Lys-AMCA into *C. elegans* and to confirm the data of the RNAi screen, a Leica TCS SP2 Confocal System coupled to a DM IRB microscope (Leica, Germany) was used. To confirm the RNAi screen data, rrf*-3(pk1426)* L4 larvae were treated with dsRNA of one of the 33 RNAi clones selected from the screen, washed with M9 buffer and then incubated with 1 mM ß-Ala-Lys-AMCA for three hours. To analyze the impact of peptidase inhibitors and amino acid supplementation on PEPT-1 function, L4 larvae (*rrf-3*;vc(RNAi), *rrf-3*;ZC416.6(RNAi) and *rrf-3*;R11H6.1(RNAi)) were washed off agar plates with M9 buffer and then washed twice. The nematodes were co-incubated with 0.01, 0.1 and 1.0 mM bestatin or amastatin or amino acids (1∶10 dilution of 1∶1 mixture of 100× non essential amino acids and 50× essential amino acids without glutamine from Minimum Essential Medium Eagle (MEM)-alpha medium (PAA, Austria)) and 1 mM ß-Ala-Lys-AMCA for three hours. After the incubation, worms were washed several times with M9 buffer, anesthetized and loaded onto an object slide. Image series were taken with 10 horizontal z-slices and the maximum fluorescence was calculated based on all sections. The fluorescence intensities of at least 10 worms per experiment were determined for the area posterior the pharynx using the Leica Confocal Software 5.2 and were normalised to the control. Each experiment was performed in duplicate.

### Labeling of *C. elegans* with a fluorescent fatty acid probe

To determine time-dependent fatty acid uptake in *C. elegans*, L4 larvae were labeled with the fatty acid analog 4,4-difluoro-5-methyl-4-bora-3a,4a-diaza-3-indacene-dodecanoic acid (BODIPY 500/510 C1,C12, Invitrogen, Molecular Probes, Germany). L4 larvae were incubated for 10 minutes with BODIPY-C12 diluted in M9 buffer to obtain the final concentration of 20 nM. (and 0.1% DMSO). Nematodes were washed before loading onto an object slide. The BODIPY fluorescence was visualized using the Leica TCS SP2 Confocal System coupled to a DM IRB microscope (Leica, Germany).

### Fat staining

For the visualization of intestinal and hypodermal fat in *C. elegans,* Sudan Black B staining was employed and prepared according to an established protocol [19]. The black-blue stained fat granules were visualized with a Leica DM IRB microscope (Leica, Germany) coupled to a digital camera. The experiment was performed twice independently and each time the diameter of 140–200 fat droplets per RNAi clone was measured. A *pept-1* like phenotype was considered, when the mean fat droplet diameter was comparable to or even higher than that measured in *rrf-3;pept-1*(RNAi) worms.

### RNA preparation and cDNA synthesis

Synchronized L1 larvae were grown on dsRNA-producing bacteria until they reached the L4 larval stage. Total RNA was isolated from each *C. elegans* sample using a combination of TRIZOL® (Invitrogen, Germany) extraction till the ethanol precipitation step followed by purification with the RNeasy Mini Kit (Qiagen, Germany). Total RNA was reverse transcribed using the Transcriptor High Fidelity cDNA Syntheses Kit (Roche, Germany).

### Realtime RT-PCR

Quantitative realtime RT-PCR (qPCR) was performed in a LightCycler (Roche, Germany) using the FastStart SYBR Green Kit (Roche, Germany) with 12.5 ng of cDNA per PCR reaction . Cycle parameters were as follows, annealing at 60°C for 10 s, elongation at 72°C for 20 s and melting at 95°C for 15 s. The following primer pairs were used: *ama-1*: for_5′-GTGCCGAGACAACTCATC-3′, rev_5′-GAGTCTGGATGGGTACTG-3′; *pept-1*: for_5′- ACTATGGAATGAGAACGGT-3′, rev_5′- CTTGTCCGATTGCGTAT-3′. For each sample two replicates per experiment were performed. The experiment was performed twice with independent biological samples. The primer efficiency was calculated using the LinRegPCR Interface [20]. Final analysis and normalization of the results were done by a qPCR software [21].

### P*pept-1*::gfp expression analysis

To investigate *pept-1* expression, the *pept-1* promoter-GFP *C. elegans* reporter strain BR2875 was used [10]. GFP fluorescence in L4 larvae of the F1 generation grown on the corresponding RNAi bacteria was individually visualized by a series of 10 vertical z-sections using a Leica TCS SP2 Confocal System coupled to a DM IRB microscope (Leica, Germany). The maximum fluorescence was calculated based on all sections using the Leica confocal software.

### Protein extraction from *Xenopus laevis* oocytes

To obtain PEPT-1 in higher yield, *Xenopus* oocytes were injected with *pept-1* cRNA [20]. Five days after the injection, the oocytes were homogenized in equal volumes of lysis buffer (20 mM HEPES, 10 mM KCl, 1.5 mM MgCl_2_, 1 mM DTT and 5 µl PMSF). The solution was centrifuged for 5 minutes (9000× g at 4°C) and the supernatant was used as a PEPT-1 positive control for western blot analysis.

### Membrane protein extraction and protein detection

100 µl of membrane extraction buffer (0.1 M Tris/HCl, 0.5 M NaCl, 12.5% glycerol, 2 µM EDTA and 1.25 mM DTT) and glass beads (∼0.4 mm diameter) were added to 100 µl of worm pellet and the samples were homogenized for 30 seconds at 4°C for a total of 5 times (Fast Prep FP120, Thermo Savant, USA). The homogenized samples were then centrifuged for 2.5 minutes at 10000 × g at 4°C. Supernatants obtained were additionally centrifuged for 45 minutes at 20000 × g at 4°C. The resulting pellets were resuspended in 50 µl storage buffer (100 mM NaCl, 10 mM HEPES-Na, 150 mM EDTA, 1 mM DTT, 1∶500 mPIC) and the protein concentration was determined by Bradford assay. Loading buffer (8% SDS, 20% glycerol, 20% mercaptoethanol, 0,4% bromphenolblue, 250 mM Tris/HCl) was added to the samples in a 1∶4 ratio and denatured at 42°C for 2 minutes following which the membrane proteins were separated by SDS-PAGE and blotted onto a nitrocellulose membrane. After a 1 hour incubation in 5% blocking solution (1× PBS, 1% Tween 20, 5% skimmed milk powder), the membranes were incubated over night with the primary antibodies (customized rabbit anti-PEPT-1 (Pineda, Germany), rabbit anti-ATGP-1 (1∶500 dilution), rabbit anti-ATGP-2 (1∶500 dilution) (gift from F. Verrey, Switzerland), anti-PGP-2 (1∶1000 dilution) (gift from G. Hermann, USA) or goat anti-ß-Actin (1∶1000 dilution; Santa Cruz, USA)). The customized anti-PEPT-1 antibody recognizes the C-terminal peptide, NH_2_-CKGFHPDEKDTFDMHF-COOH of PEPT-1. Serum was collected 130 days post-immunization of the rabbit and was monospecifically enriched. The monospecific anti-PEPT-1 antibody was used in a 1∶5000 dilution. Membranes were washed 30 minutes and then incubated with horseradish peroxidase(HRP)-coupled secondary antibody (goat anti-rabbit IgG-HRP (1∶10000 dilution), rabbit anti-goat IgG-HRP (1∶10000 dilution) (Sigma, USA)). After several washing steps with PBS, ECL solution 1 and 2 were added and the chemiluminescence was detected with a radiographic film.

### Body length measurements

Synchronized L1 larvae were transferred to NGM agar plates containing dsRNA-producing bacteria and photographed every 24 hours until they reached adulthood. The body size of a minimum of 8 nematodes per group was calculated using the program Motic Images Plus 2.0 (Motic, China). The experiment was performed in duplicate independently.

### Number of progeny

36 L4 larvae of each experimental group were separated on 12-well NGM agar plates containing dsRNA-producing bacteria. The hatched larvae from each individual nematode were counted. The experiment was performed in duplicate independently.

### Cell culture, siRNA transfection, expression and activity of PEPT1

The TC7 subclone of human colon carcinoma cells, Caco-2/TC7 (gift from E. Brot-Laroche, 20) were grown in Dulbecco's modified Eagle's medium with 4.5 g/l glucose (DMEM high glucose; PAA, Linz, Austria) and supplemented with 15% FCS Gold, 1% non-essential amino acid solution and 1% Penicillin/Streptomycin antibiotics (PAA, Linz, Austria). The MISSION® siRNAs for human Pept1, Cndp2, Lta4h and Gpx4 were purchased in pairs from Sigma, Germany. The corresponding siRNA sequences are summarized in Table S2 in the supplementary data. Caco-2/TC7 cells were grown for 24 hours in FCS- and antibiotics-free DMEM medium until they reached 50-60% confluency. The siRNA transfection (35 or 70 nM of each pair) was performed with Lipofectamine 2000 (Invitrogen, Germany) for 72 hours following the manufacturer's instructions. The solution was replaced by DMEM without antibiotics and cells were harvested after three additional days to allow cell differentiation. mRNA extraction, cDNA synthesis and real-time RT-PCR were performed as described earlier. For the analysis of PEPT1 transport activity, Caco-2/TC7 cells cultured in 24-well plates were washed with Mes-Tris-Buffer pH 6.0 (MTB) and incubated with 500 µl MTB containing 20 µM [^14^C]Gly-Sar (GE-Healthcare, Germany). After 10 minutes, uptake was stopped by washing the cells with ice cold MTB and cells were harvested in 5% Igepal lysis buffer (pH 8.0). 3 ml scintillation cocktail (Roth, Germany) was added to the cell lysate and the radioactive signal was detected using a liquid scintillation analyzer (Perkin Elmer, Germany).

### Statistical analysis

Statistical analysis was performed by using GraphPad Prism 4.01. The Students *t*-Test was used to analyze differences between a treatment group and the corresponding control. To calculate significances between different treatment groups One-way ANOVA with Turkey post test was used.

## Results

### RNAi silencing of four genes affects PEPT-1 function

**Figure 1:**
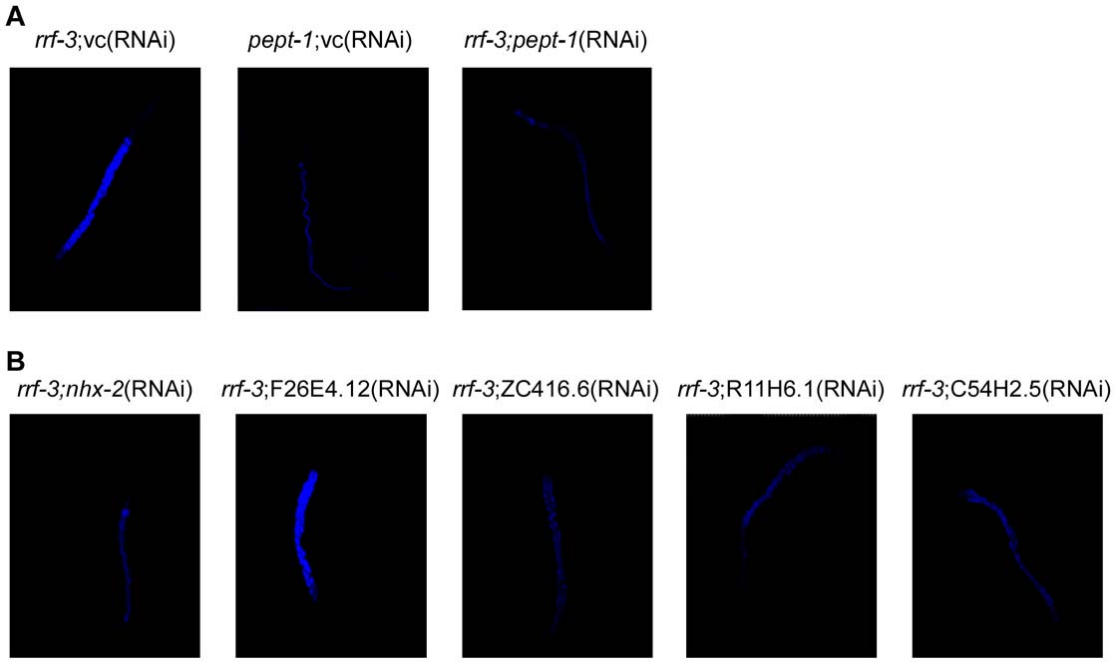
Uptake of the fluorescent dipeptide ß-Ala-Lys-AMCA. (A) ß-Ala-Lys-AMCA uptake in control *C. elegans* (*rrf-3(pk1426);*vc(RNAi), *pept-1(lg601);*vc(RNAi), *rrf-3(pk1426);pept-1*(RNAi)). (B) Panel of RNAi constructs that caused a *pept-1(lg601*)-like low ß-Ala-Lys-AMCA accumulation, on the contrary RNAi silencing of F26E4.12 induced an increased dipeptide uptake. The anterior end of the worms is located at the top of the image. All images are fluorescent overlays of 10 z-slices at a magnification of 20-fold and represent typical results.

As *pept-1* is exclusively expressed in the intestine and our focus was on the identification of modulator proteins in this tissue, the gene selection for the screen was based on data provided by Pauli et al. [22], which showed 162 genes expressed specifically in intestinal cells proven by GFP-fusion protein expression (supplementary Table S1). For assessing PEPT-1 transport function *in vivo,* the accumulation of the dipeptide ß-Ala-Lys-AMCA was analyzed. ß-Ala-Lys-AMCA is a fluorophore-conjugated dipeptide derivative which is slowly hydrolyzed and was shown to be a PEPT-1 substrate [23]. In *pept-1(lg601) C. Elegans,* the transport of ß-Ala-Lys-AMCA is completely abolished [10]. The uptake screen was performed with *rrf-3(pk1426)* worms grown on dsRNA-producing bacteria of the preselected 162 genes with fluorometric quantification of reporter substrate uptake. Gene silencing of 33 genes caused a significant (p<0.001) decrease in the uptake of the fluorescent dipeptide (supplementary Table S1), whereas only F26E4.12(RNAi) revealed an increased PEPT-1 transport function. The results of the uptake assay were independently confirmed by microscopic analysis of ß-Ala-Lys-AMCA uptake in *rrf-3(pk1426)* worms that were individually fed on dsRNA of each of the 34 potential candidate genes (Fig. 1 shows a selection). At this level, the gene silencing of 11 genes changed the ß-Ala-Lys-AMCA uptake. As a proof-of-concept, we would like to add that *nhx-2,* which was demonstrated previously to be essential for PEPT-1 function [11], [24] was one among them.

Previous work conducted by our group showed that *pept-1(lg601)* deficient *C. elegans* accumulate enormous amounts of body fat [12]. Taking this fact into account, other phenotypic features of *pept-1(lg601*)-deficient *C. elegans*, such as enlarged fat droplet size and increased fatty acid absorption were analyzed in the worms that were treated with dsRNA of the 11 candidate genes. For five genes (ZC416.6, R11H6.1, C54H2.5, F54C9.7, and C02A12.4) , fat droplet size was increased and for the other four genes (ZC416.6, R11H6.1, C54H2.5, F54C9.7), absorption of the fluorescent BODIPY-C12 fatty acid was higher than in control worms, and therefore showed *pept-1(lg601)-*like phenotypic changes (Table 1). Hence, the stepwise selection finally revealed the four candidate genes as ZC416.6, R11H6.1, C54H2.5 and F54C9.7 that when silenced caused a *pept-1(lg601)*-like phenotype with reduced dipeptide uptake, increased fatty acid absorption and body fat content. Additionally, it was found that silencing of one gene (F26E4.12) increased PEPT-1 transport activity. F54C9.7 was not taken into further analysis as it encodes a nematode-specific protein. The other four genes were further characterized to investigate their role in PEPT-1 transporter expression and function. To verify the clones from the RNAi library, the four gene constructs were sequenced and shown to possess fragments of the genes of interest (ZC416.6, R11H6.1, C54H2.5 and F26E4.12) (data not shown).

**Table 1:** Selection of putative PEPT-1 modulators.

Worm strain	RNAi	Gene name	Relative ß-Ala-Lys-AMCA uptake	Fat droplet diameter [µm]	BODIPY-C12 uptake
**controls**					
*rrf-3(pk1426)*	vector control		0.98±0.03	1.368 +++	normal
*pept-1(lg601)*	vector control		0.00±0.00 ***	3.401 +++	increased
*rrf-3(pk1426)*	K04E7.7	*pept-1*	0.13±0.04 ***	2.148	increased
**putative PEPT-1 modulators**					
*rrf-3(pk1426)*	B0495.4	*nhx-2*	0.45±0.08 ***	1.099 +++	decreased
*rrf-3(pk1426)*	B0041.5		0.37±0.08 ***	1.155 +++	normal
*rrf-3(pk1426)*	F13G3.9	*mif-3*	0.41±0.03 ***	1.501 +++	normal
***rrf-3(pk1426)***	**F26E4.12**		2.95±0.43 ***		
***rrf-3(pk1426)***	**F54C9.7**		0.47±0.06 ***	**2.221**	**increased**
*rrf-3(pk1426)*	F56C9.7		0.03±0.02 ***	1.701 +++	normal
***rrf-3(pk1426)***	**ZC416.6**		0.58±0.04 ***	**2.341 ++**	**increased**
*rrf-3(pk1426)*	F11E6.5	*elo-2*	0.30±0.09 ***	1.973 +	normal
*rrf-3(pk1426)*	C02A12.4	*lys-7*	0.61±0.05 ***	2.672 +++	normal
*rrf-3(pk1426)*	C13C4.5		0.37±0.11 ***	1.863 +++	normal
***rrf-3(pk1426)***	**R11H6.1**	***pes-9***	0.06±0.02 ***	**2.632 +++**	**increased**
***rrf-3(pk1426)***	**C54H2.5**	***sft-4***	0.41±0.03 ***	**2.201**	**increased**

Twelve putative PEPT-1 modulators were preselected by significantly altered ß-Ala-Lys-AMCA uptake, while as an exception F26E4.12(RNAi) induced an increased PEPT-1 transporter activity. To further test for a *pept-1(lg601)-*like phenotype the body fat content (based on fat droplet diameter) and the free fatty acid uptake were additionally investigated. In case of the fat droplet diameter, a *pept-1*-like phenotype was considered, when the mean diameter was comparable to that measured in *rrf-3;pept-1*(RNAi) worms or even higher, whereas the BODIPY-C12 fatty acid uptake had to be increased when compared to *rrf-3;vc*(RNAi). RNAi silencing of four genes changed all three parameters in a *pept-1(lg601)-*like manner. Statistical analysis was performed by Student's *t*-Test. Significance to *rrf-3*;vc(RNAi) (*** p<0.001) and to *rrf-3;pept-1*(RNAi) (+p<0.05, ++p<0.01 and +++ p<0.001) is denoted.

### Detailed phenotypic analysis after RNAi silencing of the modulators

#### Postembryonic growth and reproduction

**Figure 2:**
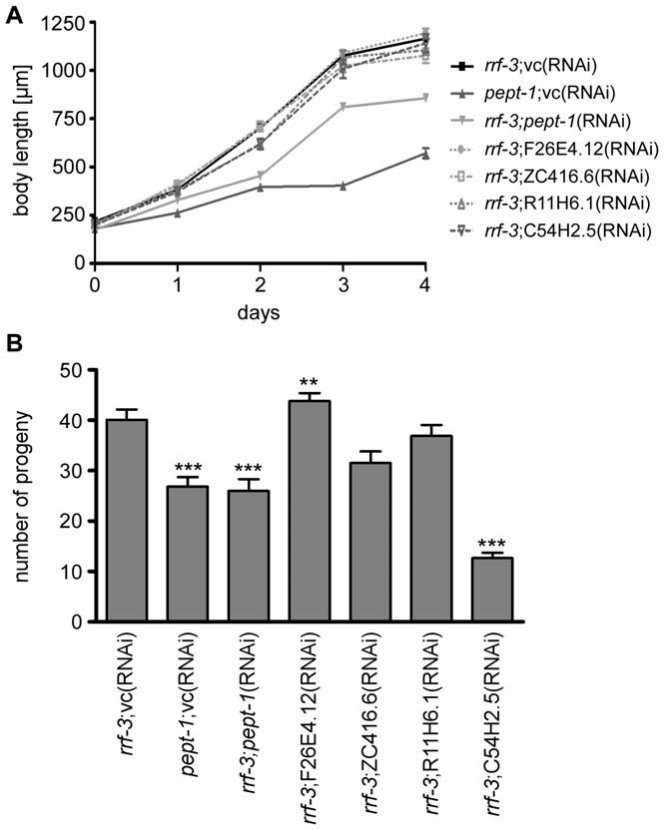
Effect of PEPT-1 modulators on larval growth and reproduction. (A) Larval growth of *C. elegans* with RNAi gene silencing of PEPT-1 modulators and controls. Daily (24 hour period), eight developing larvae per group were photographed and their body length measured. (B) Number of progeny of control worms and *rrf-3(pk1426)* worms after RNAi silencing of PEPT-1 modulators. For each RNAi construct, the progeny of 28 to 35 individual worms was counted. Each bar represents the mean ± SEM. Statistical analysis was performed by Student's *t*-Test. Significance (** p<0.01, *** p<0.001) to *rrf-3*;vc(RNAi) is denoted.

*C. elegans* lacking PEPT-1 show retarded postembryonic growth and reproduction [10]. The effects of the gene silencing of the four genes on these phenotypic characteristics were analyzed. Examination of the postembryonic growth in *rrf-3*;vc(RNAi) worms revealed an adult body length of 1164±20 µm, which is reduced by about 40% in *pept-1*;vc(RNAi) and *rrf-3;pept-1*(RNAi) *C. elegans* (Fig. 2A). Nevertheless, gene silencing of F26E4.12, ZC416.6, R11H6.1 or C54H2.5 in *rrf-3(pk1426) C. elegans* did not alter adult body length. When examining the number of progeny, we found that silencing of ZC416.6 and C54H2.5 significantly reduced reproduction to a degree that was similar to *pept-1*;vc(RNAi) and *rrf-3*;*pept-1*(RNAi) *C. elegans* (Fig. 2B). At this point, it has to be stressed that the reproduction rate of *rrf-3(pk1426)* worms on vector control RNAi bacteria is strongly reduced when compared to *rrf-3(pk1426)* grown on *E. coli* OP50 bacteria (40±2 versus 250±10 hatched larvae), an effect that was also reported by Brooks et al. [25].

#### *pept-1* promoter activity and mRNA expression

**Figure 3:**
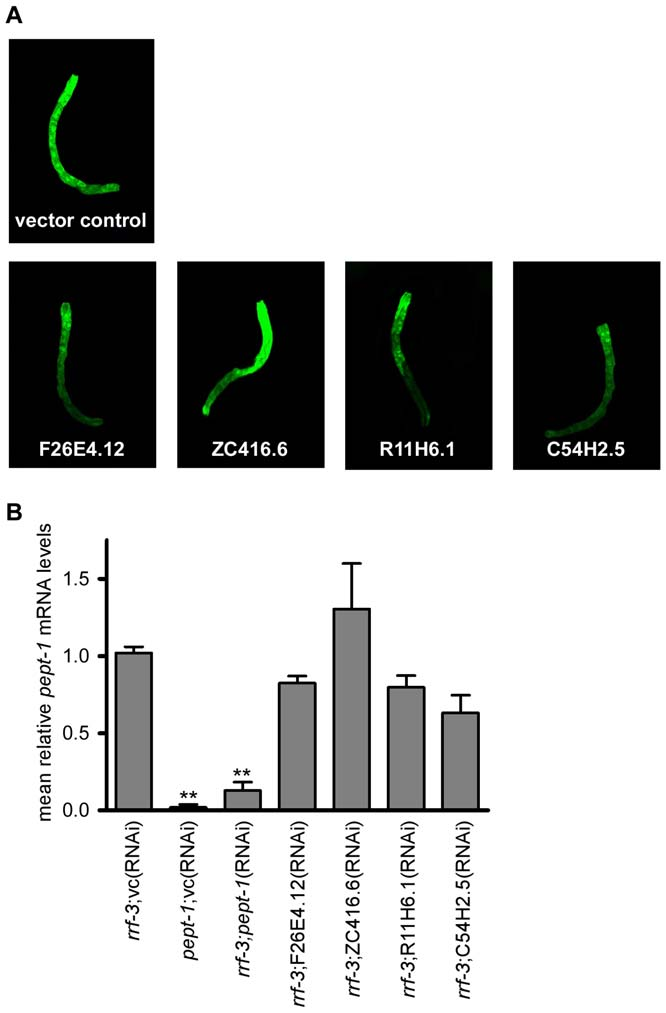
Impact of PEPT-1 modulators on *pept-1* promoter activity and mRNA expression. (A) P*pept-1*::GFP *C. elegans* treated with RNAi of the four selected PEPT-1 modulators and controls. All images are fluorescent overlays of 10 z-slices at a magnification of 40-fold and represent typical results. The experiment was performed twice, each time with images of ten individual worms. The anterior end is located at the top of the image. (B) Mean *pept-1* mRNA expression in control worms and *C. elegans* with RNAi knockdown of F26E4.12, ZC416.6, R11H6.1 and C54H2.5. The experiment was performed independently twice each time with three replicates. Each bar represents the mean ± SD. Statistical analysis was performed by a Student's *t*-test. Significance (** p<0.01) to *rrf-3*;vc(RNAi) is denoted.

The influence of RNAi silencing of the four genes on *pept-1* promoter activity was determined by using P*pept-1*::GFP worms with a *pept-1* promoter driven GFP expression in intestinal cells. Gene silencing of F26E4.12, R11H6.1 and C54H2.5 RNAi resulted in a slight reduction of *pept-1* promoter activity, whereas silencing of ZC416.6 slightly increased the fluorescent signal (Fig. 3A). In *pept-1*(*lg601*) *C. elegans,* no specific *pept-1* mRNA was detectable by real-time RT-PCR (Fig. 3B). Treatment with *pept-1*(RNAi) decreased *pept-1* mRNA concentration in *rrf-3(pk1426) C. elegans* by approximately 15% of that in control worms. RNAi silencing of F26E4.12, R11H6.1 and C54H2.5 caused a slight reduction of *pept-1* mRNA whereas ZC416.6(RNAi) slightly increased it. Although these data were not significant, the changes appear in line with the observed changes in *pept-1* promoter activity.

#### PEPT-1 protein expression

**Figure 4:**
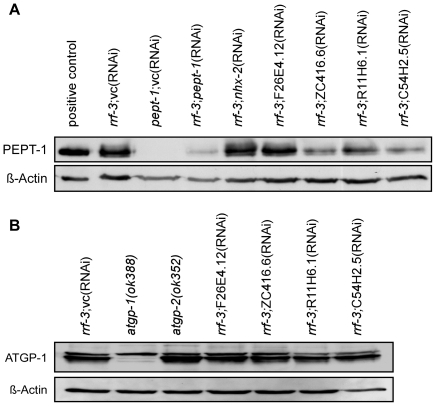
PEPT-1 protein expression is selectively altered by RNAi silencing of the modulators. Expression of selected membrane proteins in *rrf-3(pk1426) C. elegans* treated with RNAi of the controls and of the four PEPT-1 modulators. (A) PEPT-1 protein expression. 20 µg membrane protein lysate was loaded per lane. Oocytes expressing *C. elegans* PEPT-1 were used as a positive control. (B) ATGP-1 protein expression. 30 µg membrane protein lysate was loaded per lane. In both cases ß-Actin was used as a loading control.

Western blot analysis with an anti-PEPT-1 antibody was performed to determine the effects of RNAi gene silencing of the four genes on PEPT-1 protein expression (Fig. 4A). The PEPT-1 protein when expressed in *Xenopus laevis* oocytes served as a positive control. *C. elegans* membrane protein lysates included epithelial membrane bound proteins, cytoskeletal proteins including ß-actin and proteins localized to transport vesicles, which was proven for the two membrane proteins PEPT-1 and ATGP-2. These two proteins could be visualized only in the membrane fraction and not in the cytosolic fraction (data not shown). In *pept-1*;vc(RNAi) worms, no PEPT-1 protein was detected, whereas a low protein expression was observed for *rrf-3*;*pept-1*(RNAi). RNAi gene silencing of *nhx-2* and F26E4.12 did not alter PEPT-1 protein expression, while gene silencing of ZC416.6, R11H6.1 and C54H2.5 caused decreased PEPT-1 levels. As these changes are not in line with the mRNA expression of *pept-1* (see Fig. 3B) it might be suggested that they are driven by post-transcriptional processes.

### Modulator gene/protein characteristics

As the characteristics of the mammalian homologues of the selected *C. elegans* PEPT-1 modulator proteins are diverse and they influence various cellular processes, each candidate was analyzed individually. How these proteins may be linked to PEPT-1 function was assessed by additional biochemical and physiological measurements.

#### Dipeptide uptake is enhanced by RNAi silencing of a phospholipid hydroperoxid glutathione peroxidase

The gene F26E4.12 codes for a homologue of the mammalian phospholipid hydroperoxide glutathione peroxidase (PHGPx, GPx4) which catalyzes the reduction of phospholipid hydroperoxides with glutathione [26] and when silenced, exclusively caused an increase in PEPT-1 transport activity. It was shown previously that the unsaturated aldehyde 4-HNE accumulates when phospholipid hydroperoxides are not degraded by GPx4, and forms 4-HNE conjugated proteins [27]. We have validated the function of F26E4.12 by measuring the cellular 4-hydroxynonenal (4-HNE) content in *C. elegans* with and without F26E4.12(RNAi). By immunoblot analysis with an anti-4-HNE antibody (Millipore, Germany) we found a higher 4-HNE content in F26E4.12(RNAi)-treated worms than in controls (Supplementary Fig. S1) while the mitochondrial ROS load was not influenced (data not shown), pointing to a peroxidase function of F26E4.12 in *C. elegans* . The increased 4-HNE levels could modulate PEPT-1 function.

#### PEPT-1 is one of the proteins transported by the ER cargo protein C54H2.5

C54H2.5 (*sft-4)* codes for a putative ER to Golgi cargo transport protein. It is homologous to Erv29 (ER vesicle) in *S. cerevisiae* which is required for the delivery of specific secretory proteins with correct folding from the ER to the Golgi and likely acts during vesicle exit from ER [28]. As shown in Erv29Δ yeast cells, the cargo transport protein seems to control trafficking of a subset of membrane proteins while targeting and trafficking of others was not influenced [29].

We have show that silencing of the cargo transport protein encoded by C54H2.5 altered PEPT-1 protein levels, whereas another membrane protein (ATGP-1) was not affected (Fig. 4B). To assess whether C54H2.5 might also have a selective function in *C. elegans* like Erv29 in yeast, the expression of two other *C. elegans* membrane proteins namely amino acid transporter glycoprotein 2 (ATGP-2), located in the cell surface [30] and the ABC transporter homologue, PGP-2 localized to the gut granule membrane [31] was analyzed (Supplementary Fig. S2). In *rrf-3(pk1426) C. elegans, gene* silencing of C54H2.5 caused a reduced ATGP-2 protein expression without affecting PGP-2 protein levels. Our results indicate that C54H2.5 is involved in the transport of a subset of proteins including PEPT-1 and ATGP-2 from ER to Golgi. The expression of these proteins was reduced by gene silencing of C54H2.5, whereas protein expression of ATGP-1 and PGP-2 remained unaffected.

Additionally and to our knowledge for the first time, we could show that the loss of either one of the amino acid transporter glycoprotein genes *atgp-1* or *atgp-2*, both coding for the heavy subunits of heteromeric amino acid transporters, is compensated by an increased protein expression of the other isoform (Fig. 4B and Suppl. Fig. S2). This is a surprising finding, since Veljkovic and coworkers (2004) showed in *Xenopus laevis* oocytes that the light subunits AAT-1 and AAT-3 only form functional amino acid transporters with ATGP-2 (formally ATG-2) but not with ATGP-1 (formally ATGP-1) [30]. We also found that PGP-2 has a lower protein expression in *pept-1(lg601)* than in wildtype worms. PGP-2 is necessary for the formation of gut granule and hence for fat stores and directly correlates with intestinal Nile Red staining intensity. Indeed, Ashrafi and coworkers (2003) found a very low Nile Red staining intensity in *pept-1*(RNAi) worms [32], although in later studies an obesity phenotype was clearly indicated in *pept-1(lg601) C. elegans* [12], [25].

### Peptidases encoded by ZC416.6 and R11H6.1 affect PEPT-1 transport

The *C. elegans* gene ZC416.6 codes for an ortholog of the bifunctional leukotriene A4 hydrolase/aminopeptidase, LTA4H which is homologous to the mammalian isoform 1 of LTA4H. Mammalian LTA4H has two main functions. It catalyzes the final step in biosynthesis of leukotriene B_4_ (LTB_4_) [33] and functions as an aminopeptidase [34]. As *C. elegans* do not synthesize leukotrienes [35], a general aminopeptidase activity might represent the main function of ZC416.6 in worms. Also, the gene R11H6.1 (*pes-9*) is predicted to function as a zinc-dependent exopeptidase, which is homologous to the human non-specific dipeptidase 2 (CNDP2, CN2), a cytosolic peptidase with a broad range of substrates [36]. Another orthologous enzyme named Dug1 was recently identified in *Saccharomyces cerevisiae* and functions as a dipeptidase as well [37]. Hence, it is tempting to note that in the present study, RNAi silencing of two predicted cytosolic peptidases reduced *C. elegans* PEPT-1 function.

**Figure 5:**
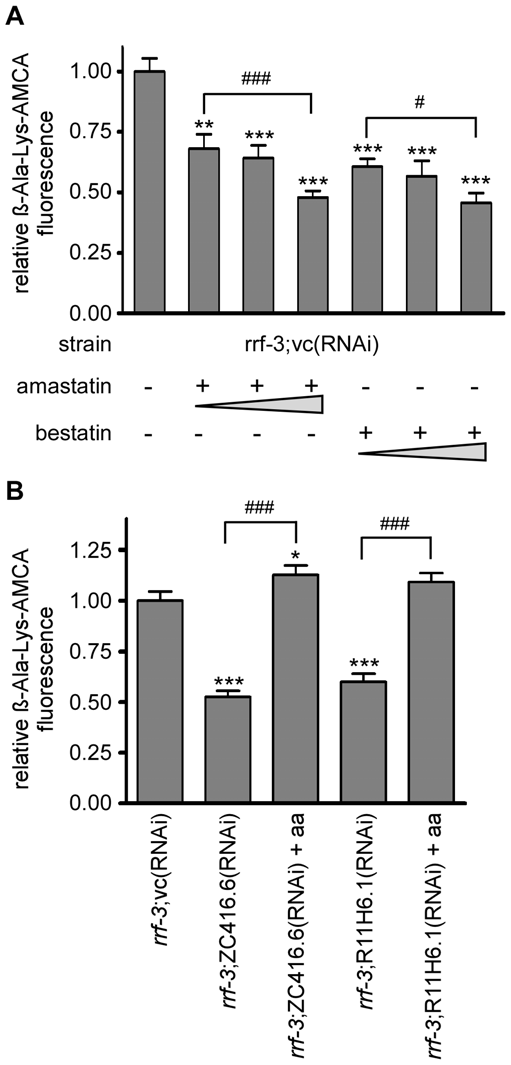
Aminopeptidase inhibition reduces ß-Ala-Lys-AMCA uptake but is compensated by amino acid supplementation. ß-Ala-Lys-AMCA fluorescence intensities of *rrf-3(pk1426) C. elegans* treated with (A) different concentrations of amastatin and bestatin and (B) RNAi of ZC416.6 and R11H6.1 with and without amino acid (aa) supplementation. The fluorescence intensities were determined for the area posterior to the pharynx. The fluorescence is denoted relative to the *rrf-3*;vc(RNAi) worms. The experiment was performed twice and each time the fluorescence of a minimum of 10 worms per group was analyzed. Each bar represents the mean ± SD. Statistical analysis was performed by a One-way-ANOVA with Turkey post test. Significance (*/# p<0.05, ** p<0.01, ***/### p<0.001) is denoted.

In extension to the work conducted on RNAi gene silencing, we applied peptidase inhibitors to assess the role of intracellular hydrolysis in PEPT-1 function. The mammalian homologues LTA4H and CNDP2 were reported to be sensitive to the aminopeptidase inhibitor bestatin [38], [39] that also serves as a substrate of peptide transporters. The general aminopeptidase inhibitor amastatin is a tetrapeptide-mimetic and has been used previously to distinguish peptide hydrolysis from transport since mammalian peptide transporters do not transport tetrapeptides [40]. *C. elegans* exposed to bestatin or amastatin showed a concentration-dependent decrease in the uptake of ß-Ala-Lys-AMCA (Fig. 5A), although peptidase inhibitor treatment did not alter PEPT-1 protein levels in lysates (data not shown). While high concentrations of bestatin could potentially inhibit ß-Ala-Lys-AMCA uptake by competition, transport inhibition at low bestatin concentrations or the amastatin effects cannot be explained by a direct action on PEPT-1. Since the peptidases could contribute to intracellular hydrolysis of di- and tripeptides entering the cells via PEPT-1, their inhibition or reduced protein levels could keep dipeptide concentrations high while decreasing the intracellular pool of free amino acids. Therefore, nematodes treated with ZC416.6(RNAi) or R11H6.1(RNAi) were supplemented with free amino acids and PEPT-1 function was determined. PEPT-1 protein levels were not affected (data not shown), but dipeptide uptake returned to levels comparable to that in control worms (Fig. 5B). This strongly suggests that the two peptidases contribute to the control of the intracellular pool of amino acids that in turn affects PEPT-1 transport capacity.

### Amino acid sensing via the TOR pathway might affect PEPT-1 protein expression and transport

**Figure 6:**
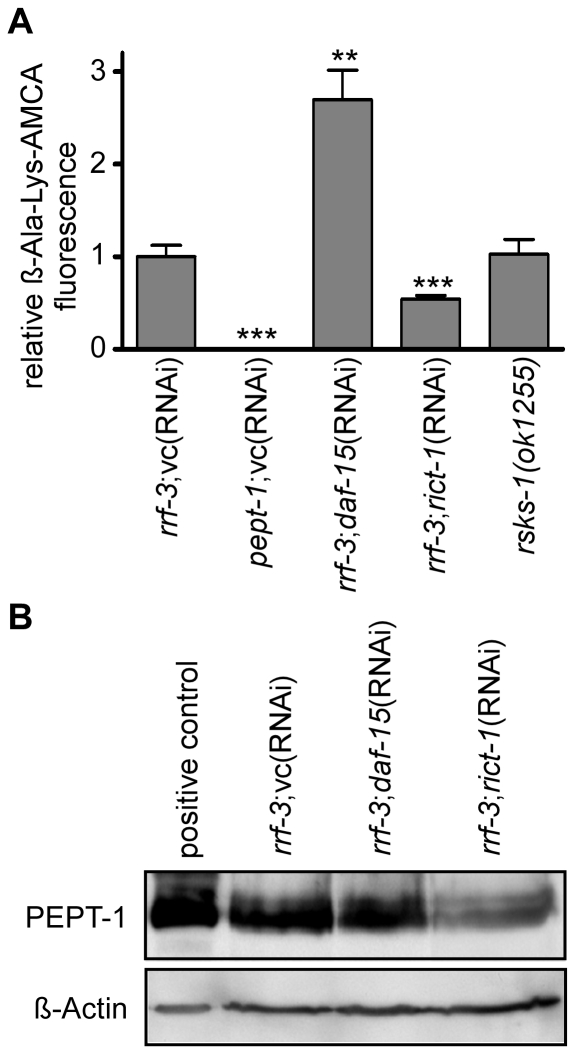
Reduced expression of genes involved in the TOR pathway alters PEPT-1 protein expression and function. (A) ß-Ala-Lys-AMCA uptake in *C. elegans* controls (*rrf-3(pk1426), pept-1(lg601)*) and in *C. elegans* with reduced expression of TOR pathway-involved genes (*daf-15*(RNAi), *rict-1*(RNAi) and *rsks-1(ok1255*)). The experiment was performed twice with four technical replicates per experiment. Each bar represents the mean ± SD. Statistical analysis was performed by a Student's *t*-Test. Significance (** p<0.01 and *** p<0.001) to *rrf-3*;vc(RNAi) is denoted. (B) PEPT-1 protein expression in controls and *rrf-3(pk1426) C. elegans* with RNAi silencing of *daf-15* and *rict-1*. 20 µg membrane protein lysate was loaded per lane. Oocytes expressing *C. elegans* PEPT-1 were used as positive control, whereas the expression of ß-Actin was used as the loading control.

It was shown that the TOR pathway acts as the main sensor of the intracellular amino acid availability [41], [42] and has a regulator function in amino acid transporter expression [43], [44]. Meissner et al. (2004) previously reported that a *pept-1* deletion intensifies the phenotype of *C. elegans* treated with weak *let-363*/TOR(RNAi), and therefore identified an upstream position of PEPT-1 to the TOR signaling pathway [10]. Since our findings suggested that the cellular free amino acid pool may participate in the control of the PEPT-1 transport capacity and that this could be mediated by TOR, we analyzed the peptide transporter expression and function in nematodes with gene defects in the TOR signalling cascade. In worms lacking the ribosomal protein S6 kinase *rsks-1(ok1255),* a target gene of the TOR pathway and essential for protein synthesis, the dipeptide uptake remained unaltered (Fig. 6A). Interestingly, treatment with *rict-1*(RNAi), a homologue of mammalian rictor and part of TORC2, reduced peptide uptake by about 50% when compared to *rrf-3(pk1426)* control (Fig. 6A), an effect due to reduced PEPT-1 protein levels (Fig. 6B). By contrast, when the expression of *daf-15*, the homologue of mammalian raptor and part of TORC1, was suppressed by RNAi silencing, dipeptide uptake was 2.8-fold higher than in the control (p<0.01) but without changes in transporter protein level. These findings strongly suggest that DAF-15 (TORC1) and RICT-1 (TORC2) participate in the control of PEPT-1 transport activity in the intestine.

### The processes regulating PEPT1 are conserved in human Caco-2/TC7 cells

**Figure 7:**
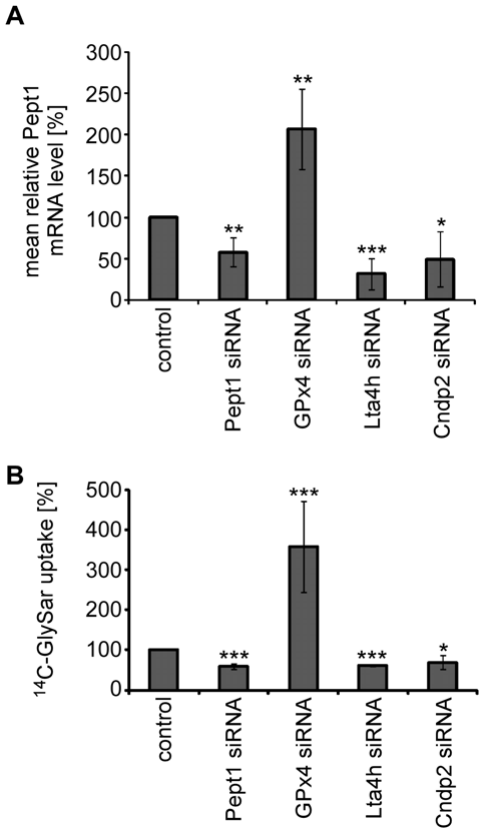
siRNA gene silencing of modulator homologues in human Caco-2/TC7 cells modulates PEPT1 mRNA expression and transporter function. mRNA expression and transporter function of PEPT1 in Caco-2/TC7 cells treated with siRNA of the modulator homologues of GPx4, Lta4h and Cndp2 relative to a siRNA control and Pept1. (A) Pept1 mRNA expression. (B) PEPT1 transporter function performed by analyzing the uptake of the radiolabelled dipeptide [^14^C]Gly-Sar. All experiments were performed at least twice with two to four technical replicates per experiment. Each bar represents the mean ± SD. Statistical analysis was performed by a Student's *t*-test. Significance (* p<0.05, ** p<0.01 and *** p<0.001) to control is denoted.

To prove that the influence of the modulators on PEPT-1 observed in *C. elegans* is also conserved in higher organisms, analysis on the homologous genes in the human colon carcinoma cell line Caco-2/TC7 was performed. The Caco-2/TC7 sub-clone is very similar to epithelial cells of the small intestine [45] and express Pept1, Cndp2, Lta4h and Gpx4 (data not shown). siRNA silencing was performed and the mRNA expression of the corresponding genes Cndp2, Pept, Gpx4 and Lta4h was reduced by 35%, 40%,70% and 90% respectively. (Supplementary Fig. S3). Contrary to the nematodes, the mRNA expression of Pept1 was significantly reduced by around 50% when the cells were treated with Pept1, Lta4h or Cndp2 siRNA, respectively (Fig. 7A). However, siRNA silencing of Gpx4 doubled the Pept1 mRNA expression. These changes were principally reflected in the PEPT1 transport activity (Fig. 7B). The results support evidence that the regulation of PEPT1 expression in human cells seem to follow another basal mechanism that already starts at the transcriptional level. Nevertheless, the final outcome in PEPT1 transporter function after gene silencing of the modulators is conserved between *C. elegans* and humans.

## Discussion

Although the intestinal peptide transporter PEPT1 has been intensively studied with respect to its kinetics, substrate specificity, dietary and pharmacological importance [for review see 5], little is known about cellular proteins that may directly or indirectly interact with the peptide transport process. In the present study, we identified four PEPT-1 modulators F26E4.12, C54H2.5, ZC416.6 and R11H6.1 in *C. elegans* with homologues in higher species that when silenced by RNA interference cause, with the exception of F26E4.12, a *pept-1(lg601)*-like phenotype.

A three-fold increased transport function without affecting *pept-1* mRNA or protein expression levels was obtained by RNAi silencing of the predicted phospholipid hydroperoxide glutathione peroxidase gene F26E4.12. The mammalian homologue PHGPx/GPx4 is one of the six isoforms of the glutathione peroxide (GPx) family which in mammals are strictly dependent on selenium as a cofactor [46]. Interestingly, in *C. elegans* only one selenoprotein, the thioredoxin reductase TRXR-1 exists [47], [48], indicating a selenium-independent function of F26E4.12. The mammalian GPx4 is a key enzyme in the protection of biomembranes exposed to oxidative stress [49] and catalyses the conjugation of phospholipid and cholesterol hydroperoxides with glutathione [46]. We show that RNAi silencing of F26E4.12 increases the concentration of the lipid peroxidation product 4-hydroxynonenal (4-HNE) in *C. elegans* confirming its predicted function. In mammals, lipid hydroperoxides initiate the activation of the transcription factor activator-protein 1 (AP-1) [50] which in turn could induce the transcriptional activation of the peptide transporter gene as shown previously [51]. The three-fold increased mRNA expression of Pept1 in human Caco-2/TC7 cells with siRNA gene silencing of GPx4 might be explained by an AP-1 dependent mechanism. Nevertheless, our data indicate that the mechanism seems not to be conserved in *C. elegans*, as in *rrf-3*;F26E4.12(RNAi) worms the *pept-1* mRNA concentration was comparable to the one in wildtype worms. The postembryonic growth and reproduction of F26E4.12(RNAi) treated nematodes were like in the wildtype. Hence, an increased function of PEPT-1 in contrast to a reduced activity does not alter the wildtype phenotype.

The ER to Golgi cargo transport protein encoded by C54H2.5 appears to participate in protein secretion, maturation and in the unfolded protein response [52]. Trafficking of PEPT-1 from ER to Golgi and to its final membrane destination may, as our findings suggest, depend on proper function of C54H2.5. Homologous genes to C54H2.5 are conserved in a number of species ranging from *S. cerevisiae* to *H. sapiens*. The best investigated homologue is Erv29p of *S. cerevisiae* [28]. As demonstrated, the amino acid transporter protein, ATGP-2 showed as well a distinct reduction in protein levels in *rrf-3;*C54H2.5(RNAi) worms, while the expression of two more membrane proteins namely ATGP-1 and PGP-2 was not altered. Therefore, the C54H2.5 protein, like Erv29p in yeast [33], seems to control trafficking of a subset of proteins leaving the ER. PEPT-1 protein and the amino acid transporter subunit ATGP-2 appear to belong to this subgroup and their impaired delivery to the apical membrane may contribute to the changes in phenotypes found in the secondary screens. However, as the silencing of C54H2.5 is very likely to affect numerous other proteins besides PEPT-1, we did not further investigate its role in proper PEPT-1 function in worms.

Gene silencing of the aminopeptidase ZC416.6/LTA4H and R11H6.1/CNDP2 in *C. elegans* and Caco-2 cells drastically reduced peptide transport and was associated with decreased PEPT-1 protein levels in the nematodes. Reduced peptide transport was associated with phenotypic changes in the nematodes similar to those found in the *pept-1(lg601)* strain such as increased fatty acid uptake and fat accumulation [12]. However, neither postembryonic growth nor reproduction was reduced significantly. This could be due to, either a residual transport activity of PEPT-1 or other compensatory mechanism such as increased amino acid absorption. Evidence for the participation of intracellular peptidases in the control of peptide transport activity was independently obtained by the application of peptidase inhibitors, bestatin and amastatin. Short term treatment of worms with these inhibitors caused a dose-dependent decrease in the uptake of the fluorescent dipeptide with no changes in PEPT-1 protein levels. From these findings we may conclude that the activity of intracellular peptidases affects intestinal peptide uptake without changes in the transporter protein level, whereas a long term suppression of the peptidase expression by RNAi silencing caused decrease in PEPT-1 protein levels.

Although the presence and high catalytic activity of cytosolic peptidases with a preference for short chain peptides in intestinal cells is known for a long time, their physiological function has not been studied yet. Di- and tripeptides entering the cells via PEPT-1 are rapidly cleaved by cytosolic peptidases to free amino acids that transiently increase the intracellular amino acid pool and then leave the cell via basolateral efflux systems [1]. However, most of these basolateral transporters act as exchangers [for review see 53] and therefore amino acid efflux from intestinal cells is counterbalanced by influx of other amino acids from the extracellular space that fill up the intracellular amino acid pool. In this respect, peptidases may increase the driving force for peptide uptake by removing the substrate from the transport equilibrium and thereby contribute to the thermodynamics of the transport process. However, when the intracellular peptide hydrolysis capacity is impaired (e.g. by RNAi knockdown of ZC416.6/R11H6.1 or peptidase inhibition) the intracellular pool of amino acids is reduced in cells expressing PEPT-1 causing short chain peptides to accumulate in the cytosol. By supplementation with free amino acids, it was clearly demonstrated that the level of intracellular amino acids is crucial for proper PEPT-1 function and/or proper PEPT-1 membrane targeting. In animals with silenced peptidases, the supplementation brought the transport activity back to a level as seen in the wildtype.

As we could show that the intracellular amino acid concentration altered by peptidase knockdown or inhibition seems to have an influence on PEPT-1 function , it was obvious that we assessed the role of the amino acid sensitive TOR pathway on PEPT-1 transport capacity and protein expression. The prime role of TOR in epithelial morphogenesis and in intestinal cell functions has been demonstrated [54], [55] and amino acids such as glutamine, arginine and leucine are considered as input signals for TOR [56], [57], [58]. Hence, the TOR protein complex acts as an intracellular amino acid sensor [42], [59], [60] with the protein components TORC1 and TORC2 displaying negative reciprocal regulation [61]. A decrease in cellular free amino acid levels was shown to cause a deactivation of TORC1 which in turn impairs protein translation by dephosphorylation of S6K1 and enhances protein degradation and turnover [62]. Inhibited S6K1 in turn activates TORC2 [63]. An increased intracellular amino acid concentration induced by amino acid supplementation may thus in reverse activate TORC1 and inactivate TORC2. We observed that RNAi silencing of *rict-1* caused reduced PEPT-1 levels and impaired di- and tripeptide transport. In this context it is important to note that, as recently shown, *rict-1(mg451) C. elegans* mutants show phenotypic characteristics reminiscent of *pept-1(lg601)* animals with increased body fat, developmental delay, smaller body size and reduced reproduction [64], [65]. By contrast, silencing of *daf-15*, the antagonist of *rict-1* increased dipeptide uptake nearly three-fold, although here the PEPT-1 protein levels were not changed. Hence, the lack of DAF-15 could activate RICT-1 by negative reciprocal regulation enhancing dipeptide uptake. PEPT-1 activity was not affected by knockout of *rsks-1* (homologous to mammalian S6K) that acts downstream of TORC1 [66] and *rsks-1(ok1255)* worms do not display a CeTOR phenotype [67]. As PEPT-1, RICT-1 and DAF-15 are all expressed in intestinal cells, an interaction of the proteins in controlling PEPT-1 expression and/or function seems plausible [10], [64], [68].

**Figure 8:**
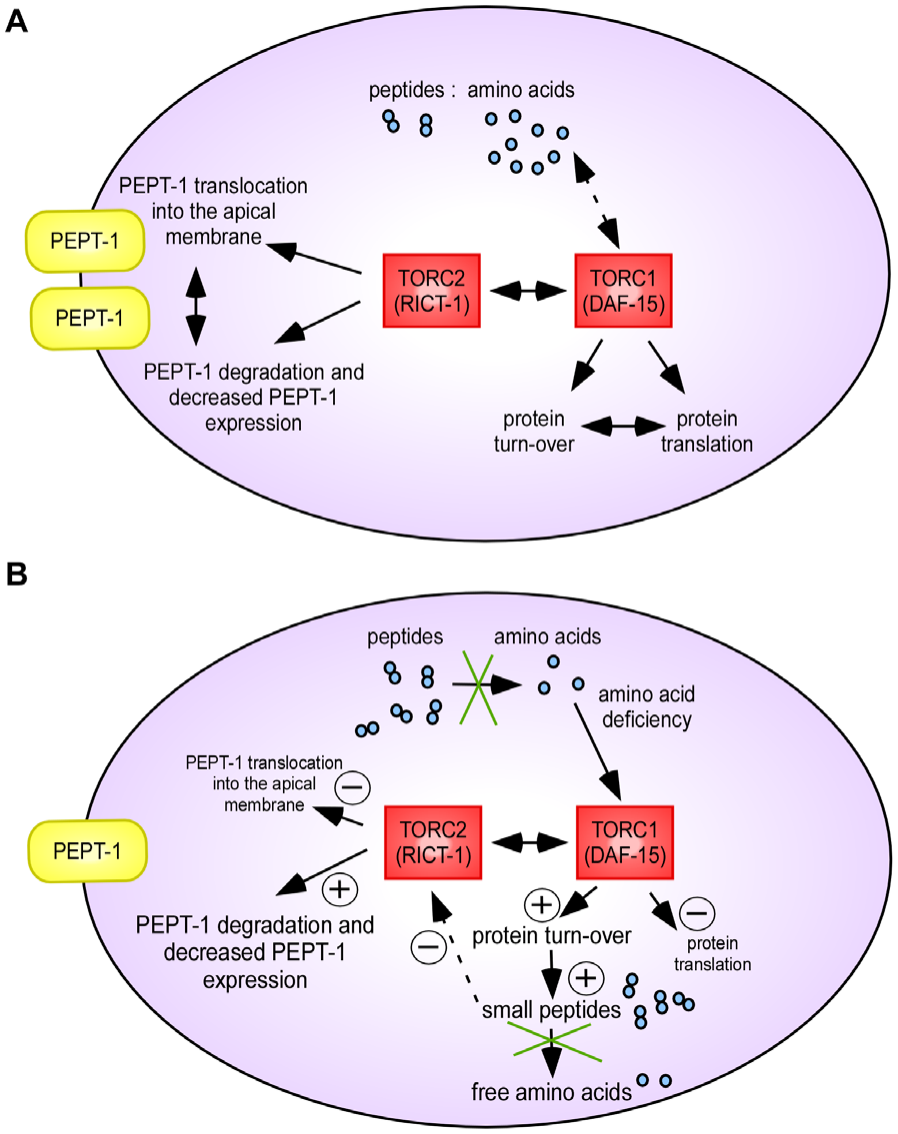
Proposed working model of the interactions between intracellular amino acid concentration, TORC1, TORC2 and PEPT-1 in *C. elegans.* (A) Model of a steady state situation in an intestinal cell of wildtype *C. elegans*. (B) Proposed altered conditions caused by RNAi gene silencing of the peptidases ZC416.6/R11H6.1 or peptidase inhibition by amastatin or bestatin. The lack of peptidases leads to an accumulation of di- and tripeptides and to a reduced concentration of free amino acids. This amino acid deficiency is detected by TORC1/DAF-15 and leads to an enhanced protein turnover with further peptide accumulation and lowered protein *de novo* synthesis. Due to the high activity of TORC1/DAF-15, TORC2/RICT-1 expression is low which is suggested to induce a retrieval of PEPT-1 from the apical membrane to the cytosolic compartment followed by its degradation.

Our data now suggest that the amino acid homeostasis in cells is indeed affected by cytosolic peptidases and that a supply-network that involves PEPT-1 may coordinate the absorption of short chain peptides, the intracellular amino acid pool and TOR signalling. On this basis, we developed a working model which displays the predicted interactions (Fig. 8). The above image demonstrates the steady state situation in the intestinal cells of *C. elegans*, whereas Figure 8B illustrates the changes caused by peptidase inhibition or RNAi knockdown of ZC416.6/R11H6.1. The lack of peptidases leads to a diminished hydrolysis of di- and tripeptides inducing a decline of the intracellular free amino acid concentration or pool. The amino acid deficiency is detected by TORC1/DAF-15 [42] and leads to an enhanced protein turnover with further peptide accumulation and a limited protein *de novo* synthesis. TORC2/RICT-1 is inhibited which is suggested to lead to retrieval of PEPT-1 from the apical membrane into cytosolic compartments with enhanced degradation as shown for PEPT1 in mammalian models [69].

In summary, we found four proteins, including two aminopeptidases that when silenced by RNAi modulate PEPT-1 function. Since the aminopeptidases most likely affect the intracellular amino acid pool that is sensed by TOR, we reasoned that PEPT-1 function might be directly influenced by the TOR pathway. Indeed we found that RICT-1 (part of TORC2) and DAF-15 (part of TORC1) change the transporter function in a reciprocal manner. A model is proposed that involves a coordinated interplay of peptide absorption, intracellular hydrolysis and translation of the amino acid pool into TOR activity affecting PEPT-1 function. Therefore, we provide evidence that the intestinal peptide transporter PEPT-1 is embedded in a complex network that regulates the cellular amino acid homeostasis in epithelial cells.

## Supporting Information

Figure S1 **Western Blot analysis of protein-bound 4-hydroxynonenal (4-HNE).** Mixed-stage cultures of *C. elegans* strains *rrf-3(pk1426)* or *pept-1(lg601)* were kept for one week on *E. coli* HT115 containing the empty vector pPD129.36 (vc) or producing dsRNA of F26E12.4 or *pept-1*. After lysis of the nematodes, 15 µg total protein was loaded per lane. 4-HNE proteins were detected with a polyclonal goat anti-4-hydroxynonenal antibody in a 1∶5000 dilution (Millipore, USA) and ß-actin was detected as loading control. In *rrf-3*;F26E12.4(RNAi) worms the signal was 15% higher than in *rrf-3*;vc(RNAi) worms, while a reduced expression of *pept-1* induced a 20–40% lower 4-HNE protein content.
(TIF)

Figure S2 **Protein expression of two additional membrane proteins altered by RNAi of the ER-cargo-transport protein.** Protein expression of two additional membrane proteins in *rrf-3(pk1426) C. elegans* treated with RNAi of controls and the modulator C54H2.5. (A) ATGP-2 protein expression of membrane protein lysates of *atgp-1(ok388)*, *atgp-2(ok352)* and of *rrf-3(pk1426) C. elegans* treated with control RNAi (vc, *pept-1*) and RNAi of C54H2.5. 20 µg membrane protein lysates were loaded per lane. (B) PGP-2 protein expression of membrane protein lysates of *rrf-3(pk1426) C. elegans* treated with control RNAi (vc, *pept-1*, and *pgp-2*) and RNAi of C54H2.5. 30 µg membrane protein lysates were loaded per lane. In both cases ß-Actin was used as a loading control.
(TIF)

Figure S3 **mRNA expression of Pept1, Gpx4, Lta4h and Cndp2 in human Caco-2/TC7 cells after siRNA silencing of the corresponding gene.** All genes show a 35 to 95 % reduced mRNA expression. Each bar represents mean ± SD and includes data from three to four independent experiments. Statistical analysis was performed by a Student's *t*-Test. Significance (* p<0.05, ** p<0.01, *** p<0.001) to siRNA control is denoted.
(TIF)

Table S1 (DOCX)

Table S2 (DOCX)
