# Supplementary figures and images for: A Glutathione Peroxidase, Intracellular Peptidases and the TOR Complexes Regulate Peptide Transporter PEPT-1 in C. elegans
Source: PLoS One. 2011 Sep 28;6(9):e25624. doi: 10.1371/journal.pone.0025624 (PMC3182239; doi:10.1371/journal.pone.0025624)

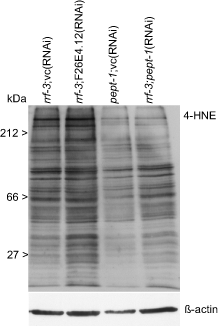

Supplement: Figure S1 — Western Blot analysis of protein-bound 4-hydroxynonenal (4-HNE). Mixed-stage cultures of C. elegans strains rrf-3(pk1426) or pept-1(lg601) were kept for one week on E. coli HT115 containing the empty vector pPD129.36 (vc) or producing dsRNA of F26E12.4 or pept-1. After lysis of the nematodes, 15 µg total protein was loaded per lane. 4-HNE proteins were detected with a polyclonal goat anti-4-hydroxynonenal antibody in a 1∶5000 dilution (Millipore, USA) and ß-actin was detected as loading control. In rrf-3;F26E12.4(RNAi) worms the signal was 15% higher than in rrf-3;vc(RNAi) worms, while a reduced expression of pept-1 induced a 20–40% lower 4-HNE protein content. (TIF) [file pone.0025624.s001.tif]

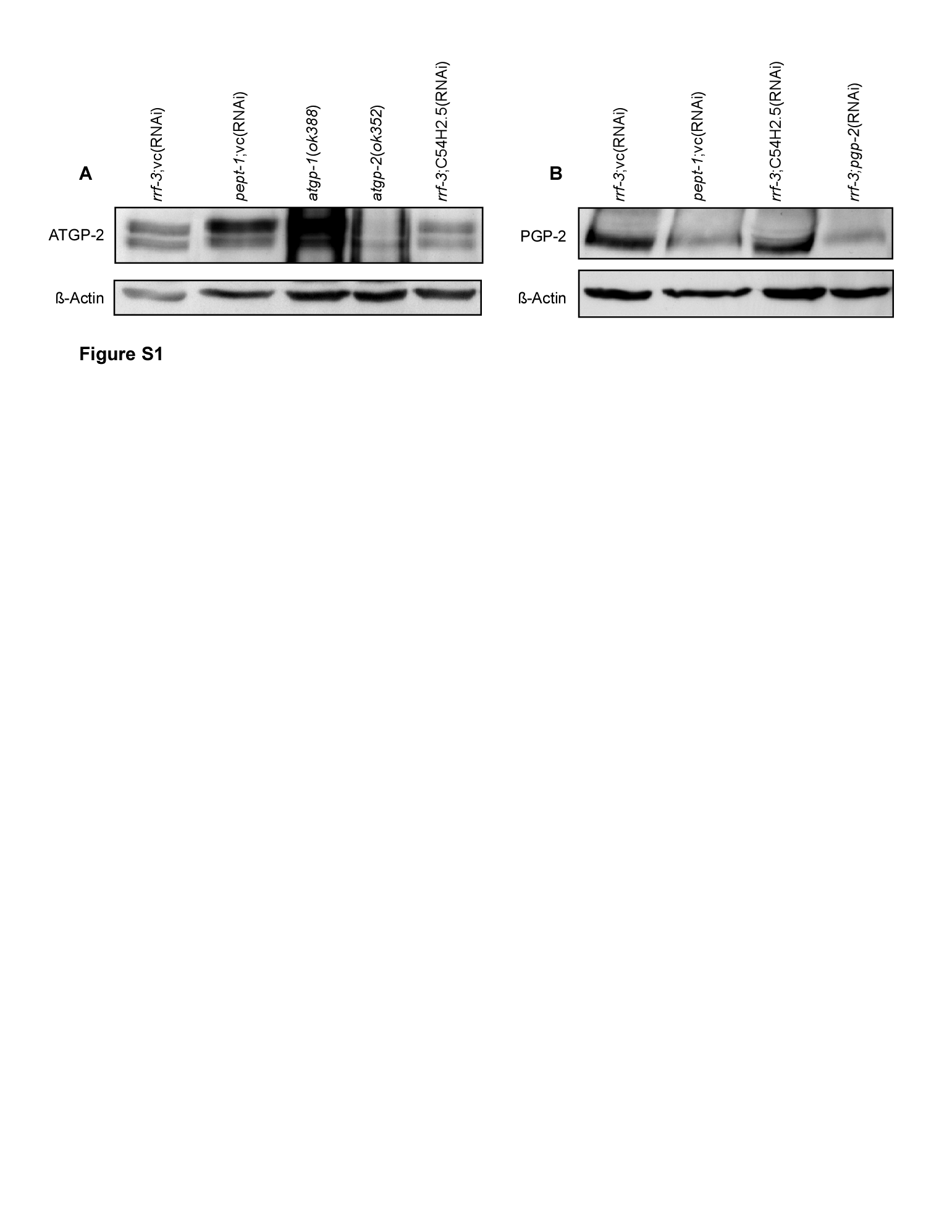

Supplement: Figure S2 — Protein expression of two additional membrane proteins altered by RNAi of the ER-cargo-transport protein. Protein expression of two additional membrane proteins in rrf-3(pk1426) C. elegans treated with RNAi of controls and the modulator C54H2.5. (A) ATGP-2 protein expression of membrane protein lysates of atgp-1(ok388), atgp-2(ok352) and of rrf-3(pk1426) C. elegans treated with control RNAi (vc, pept-1) and RNAi of C54H2.5. 20 µg membrane protein lysates were loaded per lane. (B) PGP-2 protein expression of membrane protein lysates of rrf-3(pk1426) C. elegans treated with control RNAi (vc, pept-1, and pgp-2) and RNAi of C54H2.5. 30 µg membrane protein lysates were loaded per lane. In both cases ß-Actin was used as a loading control. (TIF) [file pone.0025624.s002.tif]

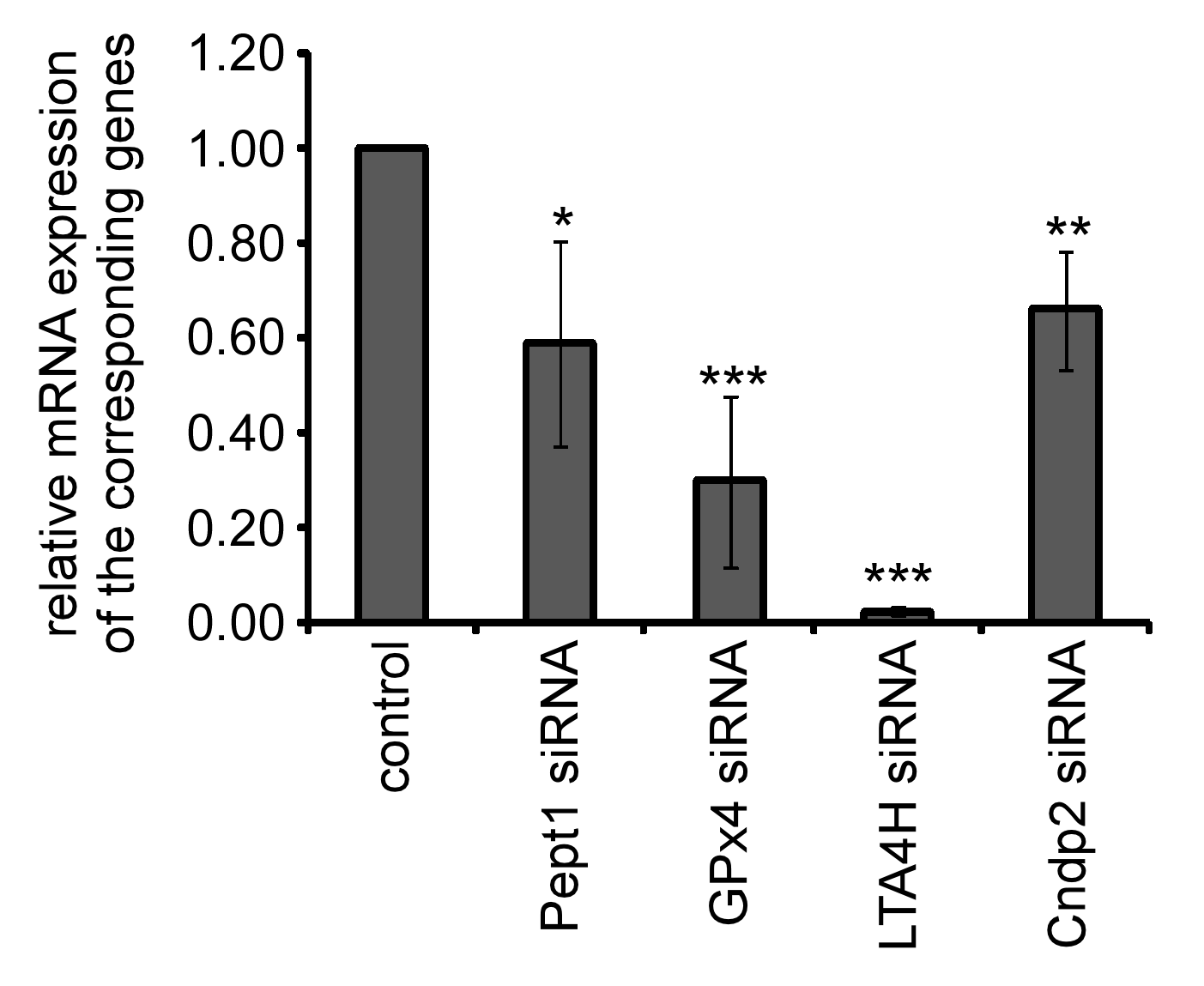

Supplement: Figure S3 — mRNA expression of Pept1, Gpx4, Lta4h and Cndp2 in human Caco-2/TC7 cells after siRNA silencing of the corresponding gene. All genes show a 35 to 95 % reduced mRNA expression. Each bar represents mean ± SD and includes data from three to four independent experiments. Statistical analysis was performed by a Student's t-Test. Significance (* p<0.05, ** p<0.01, *** p<0.001) to siRNA control is denoted. (TIF) [file pone.0025624.s003.tif]
