# Supplementary material for: A Glutathione Peroxidase, Intracellular Peptidases and the TOR Complexes Regulate Peptide Transporter PEPT-1 in C. elegans
Source: PLoS One. 2011 Sep 28;6(9):e25624. doi: 10.1371/journal.pone.0025624 (PMC3182239; doi:10.1371/journal.pone.0025624)
Supplement: Table S1 — (DOCX) [file pone.0025624.s004.docx]

**Supplementary Table S1**

List of intestinal expressed genes that were RNAi silenced and its effect on the transporter function of the intestinal peptide transporter PEPT-1.

| **Cosmid number** | **Gene** | **Chromosome** | **Relative**  **ß-Ala-Lys-AMCA uptake** | **P versus control** |
| --- | --- | --- | --- | --- |
| K04E7.7 | *pept-1* | X | 0.13 ± 0.04 | p<0.001 |
|  |  |  |  |  |
| C32E8.9 |  | I | 1.25 ± 0.32 |  |
| C34G6.4 | *pgp-2* | I | 1.18 ± 0.28 |  |
| M01A10.3 |  | I | 1.12 ± 0.28 |  |
| B0041.5 |  | I | 0.37 ± 0.08 | p<0.001 |
| F55A12.7 | *apm-1* | I | 0.52 ± 0.15 | p<0.01 |
| C24A11.8 | *frm-4* | I | 1.23 ± 0.37 |  |
| T08B2.10 | *rps-17* | I | 0.11 ± 0.08 | p<0.001 |
| F27D4.1 |  | I |  |  |
| K02F2.2 |  | I | 0.80 ± 0.07 |  |
| K06A5.6 |  | I | 0.34 ± 0.08 | p<0.001 |
| F13G3.9 | *mif-3* | I | 0.41 ± 0.03 | p<0.001 |
| R11A5.4 |  | I | 0.34 ± 0.06 | p<0.001 |
| T10B11.2 |  | I | 1.05 ± 0.05 |  |
| W01A8.2 |  | I | 1.06 ± 0.18 |  |
| F43G9.1 |  | I | 0.76 ± 0.10 |  |
| F43G9.3 |  | I | 1.01 ± 0.16 |  |
| C17E4.5 | *papb-2* | I | 0.66 ± 0.11 | p<0.01 |
| F26E4.12 |  | I | 2.95 ± 0.43 | p<0.001 |
| F46A9.5 | *skr-1* | I | 1.19 ± 0.47 |  |
| F25H2.5 |  | I | 0.80 ± 0.16 |  |
| K11D2.2 | *asah-1* | I | 1.07 ± 0.18 |  |
| Y25C1A.13 |  | II | 1.26 ± 0.43 |  |
| ZK430.2 | *tag-231* | II | 1.06 ± 0.46 |  |
| T05A7.4 | *hmg-11* | II | 0.78 ± 0.21 |  |
| C27D6.4 |  | II | 1.27 ± 0.19 |  |
| F28B12.2 | *egl-44* | II | 0.94 ± 0.29 |  |
| C17G10.5 | *lys-8* | II | 0.98 ± 0.15 |  |
| C18A3.6 | *rab-3* | II | 1.33 ± 0.25 |  |
| C32D5.2 | *sma-6* | II | 1.11 ± 0.30 |  |
| C32D5.9 | *lgg-1* | II | 1.51 ± 0.35 |  |
| T28D9.2 | *rsp-5* | II | 1.00 ± 0.37 |  |
| B0228.7 |  | II | 1.13 ± 0.13 |  |
| T05A6.1 | *cki-1* | II | 1.74 ± 0.35 |  |
| F43E2.8 | *hsp-4* | II | 1.20 ± 0.22 |  |
| C56C10.8 | *icd-1* | II | 1.23 ± 0.29 |  |
| T02G5.8 | *kat-1* | II | 1.71 ± 0.41 |  |
| C27H5.3 |  | II | 1.51 ± 0.35 |  |
| F54C9.7 |  | II | 0.47 ± 0.06 | p<0.001 |
| B0495.4 | *nhx-2* | II | 0.45 ± 0.08 | p<0.001 |
| K01C8.10 | *cct-4* | II | 1.62 ± 0.48 |  |
| K08F8.1 |  | II | 1.76 ± 0.38 |  |
| F49E12.9 |  | II | 1.13 ± 0.17 |  |
| D2013.10 | *tag-175* | II | 1.45 ± 0.33 |  |
| R03D7.1 |  | II | 1.65 ± 0.30 |  |
| B0334.4 |  | II | 1.88 ± 0.34 |  |
| F52H3.3 | *bath-38* | II | 1.61 ± 0.28 |  |
| F35C5.6 | *clec-63* | II | 0.84 ± 0.11 |  |
| F35C5.8 | *clec-65* | II | 1.27 ± 0.22 |  |
| F58G1.4 | *dct-18* | II | 1.64 ± 0.36 |  |
| F26H11.5 | *exl-1* | II | 1.36 ± 0.30 |  |
| H10E21.3 | *nhr-80* | III | 0.88 ± 0.03 |  |
| F42G9.2 | *cyn-6* | III | 1.03 ± 0.15 |  |
| F23H11.5 |  | III | 0.88 ± 0.01 |  |
| C09F5.2 | *orai-1* | III | 0.81 ± 0.10 |  |
| C36A4.9 |  | III | 0.88 ± 0.43 |  |
| C35D10.2 |  | III | 0.86 ± 0.27 |  |
| ZC395.2 | *clk-1* | III | 0.75 ± 0.19 |  |
| C35D10.14 | *clec-5* | III | 0.86 ± 0.21 |  |
| F26F4.6 |  | III | 0.74 ± 0.16 |  |
| B0285.3 |  | III | 1.01 ± 0.32 |  |
| F54D8.3 | *alh-1* | III | 0.88 ± 0.05 |  |
| R07E5.7 |  | III | 0.92 ± 0.06 |  |
| B0336.2 | *arf-1.2* | III | 1.25 ± 0.02 |  |
| B0336.7 |  | III | 1.14 ± 0.24 |  |
| B0280.3 |  | III | 0.89 ± 0.03 |  |
| F37C12.9 | *rps-14* | III | 0.39 ± 0.04 | p<0.001 |
| F01F1.2 |  | III | 0.99 ± 0.14 |  |
| F47D12.4 | *hmg-1.2* | III | 0.85 ± 0.06 |  |
| C16A3.10 |  | III | 0.81 ± 0.11 |  |
| C05D11.5 |  | III | 0.76 ± 0.17 |  |
| C05D11.10 |  | III | 1.44 ± 0.49 |  |
| F56C9.7 |  | III | 0.03 ± 0.02 | p<0.001 |
| K12H4.5 |  | III | 1.26 ± 0.14 |  |
| R13A5.8 | *rpl-9* | III | 0.37 ± 0.15 | p<0.001 |
| C30C11.2 | *rpn-3* | III | 0.73 ± 0.11 |  |
| ZK112.1 | *pcp-1* | III | 0.97 ± 0.15 |  |
| ZK652.2 | *tomm-7* | III | 0.90 ± 0.49 |  |
| ZK1098.10 | *unc-16* | III | 0.68 ± 0.21 |  |
| Y47D3B.2 | *nlp-21* | III | 0.85 ± 0.06 |  |
| Y76A2B.5 |  | III | 1.17 ± 0.05 |  |
| F53A2.4 | *nud-1* | III | 0.94 ± 0.24 |  |
| Y56A3A.18 |  | III | 0.77 ± 0.17 |  |
| Y56A3A.19 |  | III | 1.25 ± 0.13 |  |
| Y75B8A.4 |  | III | 0.76 ± 0.39 |  |
| K02D7.4 | *dsc-4* | III | 0.35 ± 0.08 | p<0.001 |
| Y66H1B.4 | *spl-1* | IV | 0.54 ± 0.09 | p<0.001 |
| ZC416.6 |  | IV | 0.58 ± 0.04 | p<0.001 |
| F49E8.4 | *cdd-2* | IV | 1.06 ± 0.02 |  |
| F49E8.5 | *dif-1* | IV | 0.67 ± 0.03 | p<0.001 |
| F33D4.1 | *nhr-8* | IV | 0.93 ± 0.03 |  |
| F59B8.2 |  | IV | 0.88 ± 0.12 |  |
| B0218.8 | *clec-52* | IV | 1.07 ± 0.15 |  |
| C49C8.5 |  | IV | 0.83 ± 0.10 |  |
| T04B2.5 |  | IV | 0.82 ± 0.09 |  |
| W08D2.4 | *fat-3* | IV | 0.88 ± 0.28 |  |
| K08F4.7 | *gst-4* | IV | 0.89 ± 0.07 |  |
| M7.1 | *let-70* | IV | 1.20 ± 0.26 |  |
| K08C7.2 | *fmo-1* | IV | 0.85 ± 0.08 |  |
| F01G10.1 |  | IV | 0.83 ± 0.18 |  |
| F01G4.1 | *psa-4* | IV | 1.16 ± 0.19 |  |
| F01G4.6 |  | IV | 0.88 ± 0.06 |  |
| F28D1.7 | *rps-23* | IV | 0.45 ± 0.10 | p<0.001 |
| K08E4.1 | *spt-5* | IV | 0.78 ± 0.14 |  |
| K08E7.1 |  | IV | 0.23 ± 0.07 | p<0.001 |
| C49C3.5 |  | IV | 0.30 ± 0.05 | p<0.001 |
| F11E6.5 | *elo-2* | IV | 0.30 ± 0.09 | p<0.001 |
| F26D10.4 | *gem-4* | IV | 0.70 ± 0.21 |  |
| F56E10.4 | *rps-27* | V | 1.06 ± 0.37 |  |
| ZK6.7 |  | V | 0.73 ± 0.07 | p<0.01 |
| C39F7.4 | *rab-1* | V | 0.19 ± 0.03 | p<0.001 |
| F39G3.1 | *ugt-61* | V | 0.37 ± 0.04 | p<0.001 |
| C18G1.5 | *hil-4* | V | 0.79 ± 0.11 |  |
| C02A12.4 | *lys-7* | V | 0.61 ± 0.05 | p<0.001 |
| CD4.2 | *crn-2* | V | 1.10 ± 0.17 |  |
| F46E10.9 | *dpy-11* | V | 0.99 ± 0.16 |  |
| K11G9.6 | *mtl-1* | V | 0.98 ± 0.20 |  |
| F52E1.13 |  | V | 0.98 ± 0.22 |  |
| T27E4.8 | *hsp-16.1* | V | 1.32 ± 0.24 |  |
| Y22F5A.4 | *lys-1* | V | 0.10 ± 0.03 | p<0.001 |
| T19B10.11 | *mxl-1* | V | 0.56 ± 0.47 |  |
| R13H4.5 |  | V | 0.65 ± 0.38 |  |
| R11D1.11 | *dhs-21* | V | 0.85 ± 0.27 |  |
| C52E4.1 | *cpr-1* | V | 0.71 ± 0.22 |  |
| C13C4.5 |  | V | 0.37 ± 0.11 | p<0.001 |
| F58E10.4 | *aip-1* | V | 0.44 ± 0.09 | p<0.001 |
| T08G5.10 | *mtl-2* | V | 0.52 ± 0.11 | p<0.001 |
| H39E23.1 | *par-1* | V | 1.12 ± 0.16 |  |
| C53A5.3 | *hda-1* | V | 0.18 ± 0.04 | p<0.001 |
| R11H6.1 | *pes-9* | V | 0.06 ± 0.02 | p<0.001 |
| F44G3.6 | *skr-3* | V | 0.19 ± 0.02 | p<0.001 |
| T03E6.7 | *cpl-1* | V | 1.41 ± 0.42 |  |
| F38A6.3 | *hif-1* | V | 0.75 ± 0.22 |  |
| R04A9.4 | *ife-2* | X | 1.10 ± 0.14 |  |
| M6.1 | *ifc-2* | X | 1.25 ± 0.15 |  |
| C44C1.2 |  | X | 0.73 ± 0.15 |  |
| C04F6.1 | *vit-5* | X | 0.73 ± 0.27 |  |
| C46C11.2 |  | X | 0.81 ± 0.56 |  |
| K10B3.8 | *gpd-2* | X | 1.48 ± 0.53 |  |
| K05B2.5 | *pes-22* | X | 1.17 ± 0.27 |  |
| M03F4.7 | *calu-1* | X | 0.79 ± 0.77 |  |
| F35C8.6 | *pfn-2* | X | 1.02 ± 0.11 |  |
| H22K11.1 | *asp-3* | X | 0.66 ± 0.08 | p<0.01 |
| C03B1.12 | *lmp-1* | X | 0.59 ± 0.02 | p<0.001 |
| C54H2.5 | *sft-4* | X | 0.41 ± 0.03 | p<0.001 |
| C07B5.2 |  | X | 1.23 ± 0.67 |  |
| F41D9.3 | *wrk-1* | X | 1.14 ± 0.11 |  |
| F15G9.1 |  | X | 1.13 ± 0.09 |  |
| R07B1.10 | *lec-8* | X | 1.20 ± 0.21 |  |
| F21G4.2 | *mrp-4* | X | 1.37 ± 0.23 |  |
| F22F1.1 | *hil-3* | X | 0.35 ± 0.04 | p<0.001 |
| F53A9.10 | *tnt-2* | X | 0.86 ± 0.09 |  |
| B0272.3 |  | X | 0.75 ± 0.09 |  |
| C33D3.1 | *elt-2* | X | 0.43 ± 0.03 | p<0.001 |
| C26F1.10 | *flp-21* | X | 0.86 ± 0.05 |  |
| M163.3 | *his-24* | X | 0.88 ± 0.09 |  |
| T22H6.2 |  | X | 0.87 ± 0.08 |  |
| C29F7.3 |  | X | 0.79 ± 0.12 |  |
| K02A4.1 | *bcat-1* | X | 0.61 ± 0.23 |  |
| C37E2.5 | *ceh-37* | X | 0.40 ± 0.13 |  |
| F48C11.3 | *nlp-3* | X | 0.20 ± 0.03 | p<0.001 |
| K05G3.3 | *cah-3* | X | 0.54 ± 0.15 | p<0.01 |
| C06G1.4 | *ain-1* | X | 0.86 ± 0.29 |  |
