# Supplementary material for: A Glutathione Peroxidase, Intracellular Peptidases and the TOR Complexes Regulate Peptide Transporter PEPT-1 in C. elegans
Source: PLoS One. 2011 Sep 28;6(9):e25624. doi: 10.1371/journal.pone.0025624 (PMC3182239; doi:10.1371/journal.pone.0025624)
Supplement: Table S2 — (DOCX) [file pone.0025624.s005.docx]

**Supplementary Table S2**

siRNA of the human gene homologues of the PEPT-1 modulators, Pept1 siRNA served as positive control.

| **Name** | **Source** | **Start** | **Sequence 5' to 3'** | **Ref Seq** |
| --- | --- | --- | --- | --- |
| **Pept1** |  |  |  |  |
| SASI_Hs01_00057727 | HUMAN | 1587 | GCUACAAUGCCAGCACAUA[dT][dT] | NM_005073 |
| SASI_Hs01_00057728 | HUMAN | 1551 | CAAUGAGUGGGAAAGUUUA[dT][dT] |  |
|  |  |  |  |  |
| **Lta4h** |  |  |  |  |
| SASI_Hs01_00143001 | HUMAN | 1725 | CGCUAAAGAUGGCAACUGA[dT][dT] | NM_000895 |
| SASI_Hs01_00143002 | HUMAN | 228 | CAAAGGACCUUACAAUAGA[dT][dT] |  |
|  |  |  |  |  |
| **Cndp2** |  |  |  |  |
| SASI_Hs01_00187296 | HUMAN | 377 | GAGAUCCCGCUCCCUCCUA[dT][dT] | NM_018235 |
| SASI_Hs01_00187297 | HUMAN | 699 | CGGAAAGACACAUUCUUUA[dT][dT] |  |
|  |  |  |  |  |
| **GPx4** |  |  |  |  |
| SASI_Hs01_00191236 | HUMAN | 521 | GUAACGAAGAGAUCAAAGA[dT][dT] | NM_002085  |
| SASI_Hs01_00050315 | HUMAN | 256 | CGGGCACAUGGUUAACCUG[dT][dT] |  |
